# Patient-reported outcome measures in knee injuries rehabilitation: A protocol for intervention

**DOI:** 10.1016/j.mex.2024.102647

**Published:** 2024-03-05

**Authors:** José Moreira, Marina Mesquita, José Flamínio, Manuel de Almeida, Bruno Delgado, Paulo Boto

**Affiliations:** a University of Évora, Évora, Portugal; b NOVA National School of Public Health, Public Health Research Centre, Comprehensive Health Research Center, CHRC, NOVA University Lisbon, Lisbon, Portugal; c Fisicontrol, Oeiras, Portugal; d Fisioportel Rehabilitation Center, Évora, Portugal; e Egas Moniz Center for Interdisciplinary Research (CiiEM), Egas Moniz School of Health & Science, Almada, Portugal; f Catholic University of Portugal, Institute of Health Sciences, Porto, Portugal; g National School of Public Health, NOVA University of Lisbon, Lisbon, Portugal

**Keywords:** Patient-reported outcome measures, Health-related quality of life, Rehabilitation, Knee injuries, PROM_R

## Abstract

Different musculoskeletal conditions affect people all over the world and were considered by the WHO to be the main cause of disability in 4 of 6 regions in 2017, with an increase in the associated burden and the impact they have on today's society. One of these conditions is related to the knee, which is associated with complex and vulnerable injuries associated with ligaments, menisci, and cartilage. After surgery, there is a reflex inhibition of motor neurons and immobilization, there is rapid atrophy and weakness in the different associated muscles, affecting proprioception, strength and muscle function, compromising quality of life. The aim of this article is to describe a protocol for a rehabilitation program after surgery for people with knee injuries. An experimental study will be carried out with 75 patients, with control and experimental groups. In both groups, initial measurements will be compared with measurements after the program, at different times. It is hoped that this study will generate significant information on rehabilitation intervention for people with knee injuries.

## Graphical abstract

**Figure:**
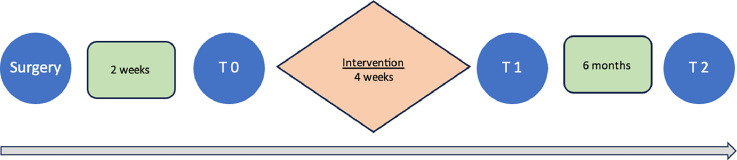

Specifications table

**Table:** 

Subject area:	Medicine and Dentistry
More specific subject area:	Rehabilitation; Health policies and research in health services.
Name of your protocol:	PROM_R
Reagents/tools:	EQ-5D-5L - EuroQol 5 Dimensions HADS - Hospital Anxiety and Depression Scale BREQ2 - Behavioural Regulations in Exercise Questionnaire
Experimental design:	An experimental study with 75 selected participants. Protocol that includes rehabilitation intervention in adults with an experimental group and a control group, enrolled in rehabilitation institutions located in rural and urban regions, carried out by the physiotherapist. Performance at the beginning of the program will be measured and then compared with performance after the supervised intervention in both groups. Participants will be included in the intervention after agreeing to take part in the study.
Trial registration:	Study protocol was registered in Clinicaltrials.gov with the number NCT06206018.
Ethics:	The study protocol was submitted to the Ethics Committee of the Nacional School of Public Health – NOVA University of Lisbon and was approved (CEENSP n°3/2022).
Value of the Protocol:	- The protocol is important for evaluating the effects of a structured four-week rehabilitation program on the quality of life reported by participants after knee injury, and who live in rural or urban regions. - There is a lack of knowledge about the selection of Patient-Reported Outcomes Measures (PROMs) considering the different domains envisaged by patients with knee pathology when participating in rehabilitation programs. - This article will contribute recommendations for a rehabilitation program based on supervised physical exercise after knee injury.

## Description of protocol

Different musculoskeletal conditions affect people all over the world and were considered by the WHO to be the main cause of disability in 4 of its 6 regions; they cause an increase in the associated burden with a real impact on our society. One of these conditions is related to the knee, which is associated with complex and vulnerable injuries, the most common of which are caused by ligaments, menisci, and cartilage. Structured rehabilitation programs are essential as they improve quality of life by optimizing proprioception, strength, and muscle function, which can be compromised immediately after surgery as a consequence of reflex inhibition of motor neurons and immobilization [1].

The aim of this experimental clinical study is to analyze the effects of a 4-week supervised rehabilitation program on the quality of life reported by participants after knee surgery. As a secondary objective, we intend to analyze the impact that the program has on the level of anxiety and motivation to practice exercise through the application of PROMs, in patients who have undergone knee surgery.

### Study design

An experimental study will be carried out, following the recommendations of CONSORT (Consolidated Standards of Reporting Trials) to assess the effectiveness of the intervention [2].

### Subjects

Participants will be selected from among patients with knee pathology who have undergone surgery, following a physiotherapy appointment.

### Inclusion and exclusion criteria

The inclusion criteria are as follows: (i) patients that are aged 18 or over; (ii) that consent to take part in the study; (iii) that can read and write; and (iv) have undergone knee surgery. The exclusion criteria are: patients with permanent or temporary dysfunctions of the central or peripheral nervous system; that have or have had previous surgeries on the ipsilateral knee; unconsolidated fractures; partial or total amputation of the upper or lower limbs.

### Sample size calculation

GPower@ software was used to calculate the sample size, determining a minimum of 60 patients assuming an alpha error of 0.05, a sample power of 0,5 (Power = 1-β err prob), and an effect size of 0,5. Anticipating a potential loss of 20% in follow-up due to the period required for the program (with evaluation at 6 months) and based on the numbers from each of the centers, a total of 75 patients will be recruited.

**Fig. 1:**
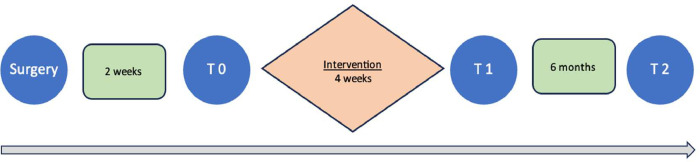
Program schedule.

The experimental group will complete the rehabilitation program, in which the intervention takes place in five sessions per week over 4 weeks, with the physiotherapist supervising all the sessions (Fig. 1).

### Recruitment

Participants will be recruited from rehabilitation clinics in an urban region (Oeiras-Portugal) and a rural region (Portel-Portugal), where they have started a recovery program. They will be invited to take part in the study immediately after their physiotherapist appointment, will be informed about the aims of the study and asked for informed consent (already approved by the Ethics Committee of the National School of Public Health - NOVA University of Lisbon).

### Procedures

Participants will complete the intervention described in Table 1, based on the updated Journal of Orthopaedic and Sports Physical Therapy (JOSPT) guidelines for knee injuries [3].

**Table 1:** Knee treatment intervention protocol.

Interventions	week			
	1	2	3	4
Massage	x	x	x	x
Passive Mobilization	x	x		
Active Mobilization	x	x	x	x
Neuromuscular Electrical Stimulation (NMES)	x	x	x	x
Proprioceptive Training		x	x	x
Isometric Exercises: Quadriceps; Vastus Medialis Oblique Muscle (VMO); Hip Adduction	x	x		
Isotonic Exercises: Gastrocnemius	x	x		
Isotonic Exercises: Quadriceps; Hip extension; Hip adduction; Hip abduction; Hamstrings	x	x	x	x
Standing Isotonic Gastrocnemius Exercises		x	x	x
Wall Slide 0° to 30°		x		
Wall Slide 0° to 45°			x	
Wall Slide 0° to 90°				x
Static Ergometer		x	x	x
Treadmill Progression: gait>run				x
Single Leg Vertical Jump				x
Specific Sport Exercises				x
Lower Limbs Stretching	x	x	x	x
Cryotherapy	x	x		

Initial data will be collected at baseline (T0), the intervention will then take place, an evaluation will be carried out shortly after the end of the intervention (T1) and the final evaluation will be carried out 6 months after the end of the intervention (T2). The following PROMs will be applied before and after the intervention:

- The “**EQ-5D-5L**” instrument comprises five dimensions, each describing a different aspect of health: Mobility, Self-care, Usual activities, Pain/discomfort, and Anxiety/depression. Each dimension has five response levels: no problems, slight problems, moderate problems, severe problems, unable to/extreme problems. The visual analogue scale of this questionnaire has a score of 0–100. Higher scores mean better quality of life and lower scores indicate lower quality of life [4];
- The “**HADS**” instrument, developed to detect symptoms of anxiety and depression, is made of 14 items, subdivided into two subscales - Anxiety and Depression, with a maximum score of 21 points for each subscale. The subscale scores can be calculated separately or together, with each item scored from 0 (absent) to 3 (extremely present) [5];
- The “**BREQ2**” instrument has 19 items with five response options on a Likert scale, where 0 is "not true for me" and 4 is "often true for me" to assess the constructs of: amotivation, external regulation, introjected, identified and intrinsic motivation. The results can vary from 24 (lowest self-determination) to 20 (highest self-determination) [6].

### Intervention description

The standard intervention is based on the physiotherapy clinical practice guidelines, updated by the JOSPT for knee injuries, which include lower limb massage, mobilization, neuromuscular strengthening and rehabilitation, teachings, quadriceps neuromuscular electrical stimulation and cryotherapy [3]. The intervention group will complete the four-week rehabilitation program, with five sessions per week, performed and supervised by the physiotherapist. All patients follow the same order of treatment and exercises, starting with massage and mobilization, followed by the corresponding exercise program, and ending with Neuromuscular Electrical Stimulation (NMES) and cryotherapy (Table 1). This four-week intervention for rehabilitation of the knee joint after surgery makes it possible to improve passive and active knee extension (knee extension range of motion), increase quadriceps strength, improve gait performance, and optimize the patient's functionality. The control group will follow a standard six-week rehabilitation program.

### Evaluation

We have three evaluations of each group: a baseline assessment, before the intervention, a 2nd evaluation right after the end of the intervention, and a 3rd, six months after the intervention.

## Discussion

This experimental clinical study makes it possible to assess the quality of life reported by participants with knee pathology who take part in a rehabilitation program after surgery. In rehabilitation after surgery, measuring results is fundamental, and focuses on assessing function, activity, and participation [7]. There is nowadays a tendency to obtain the patient's perspective through PROMs, as they include scores related to satisfaction and health-related quality of life [8]. Satisfaction and health-related quality of life are key elements in understanding the effectiveness of rehabilitation programs, making it possible to mitigate the consequences associated with knee pathology, improve the rehabilitation process, and adapt health care to the population [8].

The significant importance that patient satisfaction and quality of life has assumed in health care is in line with the construction and development of this project, promoting positive changes in health status and in the services provided through a rehabilitation program [9]. The evaluation is based on the recording of data throughout the program, focusing on the self-perception of the participants in the group with the four-week structured program and in the group with the six-week regular program.

We intend to contribute with recommendations regarding the application of PROMs used in rehabilitation programs after knee pathology, in relation to motivation, satisfaction and health-related quality of life.

## Statistical methods

The Statistical Package for Social Sciences (SPSS®) 29 software (IBM Corp, Armonk, New York, USA) will be used. Different statistical procedures will be used to analyze the data, including descriptive and inferential techniques. Non-parametric tests, Chi-square and Mann-Whitney U will be used when the normality of the distribution is not verified. If normality or small deviations allow the use of parametric tests, we will apply the Student's *t*-test for independent measures.

The differences between the groups at the different assessment times will be calculated using a two-factor ANOVA. A Multiple Linear Regression will be carried out with each dependent variable and the intervention to analyze its effect on the different outcomes found. For the statistical tests, a 95 % confidence level will be considered, even if the p-values do not indicate a significant difference, we will report the effect size to give meaning and emphasize the power of the results [10].

## Ethics statements

This study protocol was reviewed and approved by the Ethics Committee of the Nacional School of Public Health – NOVA University of Lisbon, approval number CEENSP n°3/2022. Participation in the study is voluntary, after written informed consent from all participants.

## CRediT authorship contribution statement

**José Moreira:** Conceptualization, Methodology, Software, Data curation, Writing – original draft. **Marina Mesquita:** Visualization, Investigation. **José Flamínio:** Visualization, Investigation. **Manuel de Almeida:** Visualization, Investigation. **Bruno Delgado:** Validation. **Paulo Boto:** Methodology, Supervision, Validation.

## Declaration of competing interest

The authors declare that they have no known competing financial interests or personal relationships that could have appeared to influence the work reported in this paper.

## Data Availability

No data was used for the research described in the article.
